# The efficacy and safety of oxycodone in treating the uterine contraction pain after negative pressure aspiration: A randomized, compared, multicenter clinical study

**DOI:** 10.1097/MD.0000000000030048

**Published:** 2022-08-19

**Authors:** Yi-Nan Wang, Ming-Jun Xu, Ying Feng, Xin-Zhong Chen, Fei Yao, Ming-Kun Shen, Lu-Wen Zou, Hua Feng, Lei Zhao, Jian-Guo Xu

**Affiliations:** a Beijing Obstetrics and Gynecology Hospital, Capital Medical University, Beijing Maternal and Child Health Care Hospital, Beijing, China; b Department of Anesthesiology, Women’s Hospital, School of Medicine, Zhejian University, Hangzhou, Zhejiang, China; c Department of Anesthesiology, the Affiliated Wuxi Matemity and Child Health Care Hospital of Nanjing Medical University, Wuxi, China; d Department of Anesthesiology, Xuanwu Hospital, Capital Medical University, Beijing, China; e Department of Anesthesiology, Jinling Hospital, Medical School of Nanjing University, Nanjing, China.

**Keywords:** dysmenorrhea, negative pressure aspiration, oxycodone, pain of uterus contraction

## Abstract

This study aims to evaluate the efficacy and safety of oxycodone hydrochloride in treating postoperative uterine contraction pain in dysmenorrhea patients undergoing negative pressure aspiration.

This study is a multicenter randomized controlled clinical trial. Two hundred patients with history of moderate or severe dysmenorrhea for negative pressure aspiration were divided into 4 groups evenly (n = 50): the control group (group P, isotonic saline), the low-dose oxycodone group (group O1), the medium-dose oxycodone group (group O2), and the high-dose oxycodone group (group O_3_). All the 4 groups were followed by slow (45–60 seconds) static push of propofol until eyelash reflex disappeared (a maximum dose of 2.5 mg/kg). Additional propofol (0.6–1 mg/kg) was added if body movement occurred during operation.

The postoperative VAS scores in different groups showed that there was no statistical difference between groups P and O1, while there were statistical differences when groups O2 and O3 were compared with group P (*P* < .01), and when group O3 compared with O1 (*P* < .01). Furthermore, there was no significant differences in HR and BP among the 4 groups, but the intraoperative respiratory frequency in groups O1, O2, and O3 was significantly higher than in group P (*P* < .01). The SPO_2_ after operation in groups O2 and O3 was higher than in group P (*P* < .05). There was a significant difference in the VAS ≥ 4 between group O2 or O3 and Group P (*P* < .01). The incidence of respiratory depression in P group was significantly higher than in O1, O2, and O3 groups (*P* < .01). The dilatation time and the number of dilatation bar used in group O3 is lower than that of group P and group O1 (*P* < .01). The difficulty of dilatation in group P was 26% as assessed by clinicians, with significant differences with groups O1, O2, and O3 (*P* < .01).

**Conclusion:**

Oxycodone hydrochloride injection could be safely and effectively applied to negative pressure aspiration, and a 0.08 mg/kg dose could significantly reduce postoperative uterine contraction pain of patients with dysmenorrhea.

## 1. Introduction

Nowadays, propofol, either combined with an opioid drug or without the opioid has been clinically widely applied as an effective pain-relieving anesthesia in negative pressure aspiration operation.^[1,2]^ Uterine contraction pain is a visceral pain and could not be effectively relieved by propofol lacking an analgesic effect. Even with the combined use of propofol and fentanyl, a large proportion of patients still suffer from uterine contraction pain after the artificial abortion. Previous studies show that patients with a dysmenorrhea history are the high-risk population for uterine contraction pain after negative pressure aspiration, with an incidence of over 60%.^[3]^ It is therefore highly necessary to safely and effectively resolve this adverse event in induced abortion. The oxycodone (14-hydroxy-7, 8-dihydrocodeinone) hydrochloride, a semisynthetic opioid, has been known a good analgesic for visceral pain.^[4]^ We wonder whether the use of oxycodone in combination with propofol may provide a new resolution to this problem. The aim of this study is to evaluate the efficacy and safety of using oxycodone to relieve uterine contraction pain after negative pressure aspiration, and to determine the optimal dose.

## 2. Subjects and Methods

### 2.1. Ethical approval

This study is approved by the medical ethics committee of the Beijing Maternity Hospital Affiliated to Capital Medical University (Approval No.2016-KY-048-01), and signed written informed consent document was obtained from each of the enrolled patients prior to participation. This multicentered, randomized, comparative, clinical experimental investigation was carried out by the Beijing Maternity Hospital Affiliated to Capital Medical University, the Maternity Hospital Affiliated to the School of Medicine of Zhejiang University, Wuxi Maternity and Child Health Care Hospital Affiliated to Nanjing Medical University and Xuanwu Hospital Affiliated to Capital Medical University.

### 2.2. Patient enrollment

Two hundred patients scheduled to undergo negative pressure aspiration and having dysmenorrhea history (VAS ≥ 4) were selected and the study is designed based on randomized, placebo-controlled and parallel groups. The participants are randomly divided into 4 groups: the control group (group P, isotonic saline), the low-dose oxycodone group (group O1, 0.04 mg/kg), the medium-dose oxycodone group (group O2, 0.06 mg/kg), and the high-dose oxycodone group (group O3, 0.08 mg/kg) with a ratio of 1:1:1:1. Blocked randomization method based on research center was used. Using SAS9.2 to generate the random number and SAS software package to produce the “Center code random number table,” the random coding of each center, including center’s code and the drug serial number, is obtained.

Inclusion criteria: aged 24–36 years old, with BMI between 18 kg/m^2^ to 28 kg/m^2^, ASA physical status I or II, first and singleton pregnancy (months of pregnancy 5–8), history of medium or higher degree of dysmenorrhea, operation duration within 10 minutes with the insertion of vaginal speculum as the starting time and the removal of the vaginal speculum as the ending time. Exclusion criteria: patients with obvious heart, lung or kidney dysfunction or metabolism dysfunction; with an abnormal anesthesia recovery history; allergic to propofol or oxycodone; with history of hypertension, hyperglycemia, hyperlipidemia, or alcoholism; with acute respiratory inflammation within the last 2 weeks; with oxycodone administration within 7 days prior the negative pressure aspiration; with combined use of hysterotonics by injection or oral administration, including traditional Chinese herb medicine, either before, during or after the abortion operation; suspected of abuse of anesthetic analgesics; having had or suspecting to have difficult airway; having received uterine body or cervix surgical operations or intraoperative blood transfusion. Patients meeting the inclusion criteria were enrolled in the study and signed the written informed consent document 1 day prior to the operation.

### 2.3. Anesthesia method

Monitoring systems including electrocardiography, pulse oximetry and noninvasive blood pressure measurement system were set up before anesthesia induction. Conventional mask oxygen inhalation was adopted, with an oxygen flux of 3 L/min. Open cubital vein infusion was uniformly applied and the following drugs were delivered through trocar directly connected to 3-way extension tube: for control group (group P), isotonic saline 2 mL; for low-dose oxycodone group (group O1), 0.04 mg/kg; for medium-dose group (group O2), 0.06 mg/kg; for high-dose group (group O3), 0.08 mg/kg. The oxycodone hydrochloride injection was used (BX190, BX281, BX845, BX015, Mundipharma Pharmaceutical Co., Ltd). The anesthesia was commenced by giving the above drugs during the time of 1 minute to the 4 groups respectively. All the groups were then uniformly given propofol MCT (1903042, 1906104, 1911256, 1907118, Beijing Fresenius Kabi Pharmaceutical Co, Ltd) by intravenous injection slowly and smoothly within 45 to 60 seconds until the disappearance of eyelash reflex, with a maximum dose of 2.5 mg/kg. The negative pressure aspiration was then started. During the operation, additional propofol of 0.6 to 1 mg/kg was given to the patient if BM reaction occurred.

### 2.4. Intraoperative management

During the operation, when the systolic pressure was reduced to ≤80 mm Hg in 2 successive measurements with an interval of 1 minute, ephedrine 5 to 10 mg was given by intravenous injection; When heart rate was ≤50/min and lasted for 30 seconds, atropine 0.5 mg was intravenously injected; when pulse oxygen saturation (SPO_2_) was ≤92% and lasted for 30 seconds, the lower jaw of the patient should be manually lifted up to improve the ventilation; in case the SPO_2_ was further lowered to ≤90%, simple airbag mask was used to help the respiration. The fluid intake was kept at a rate of about 10 mL/kg/h.

### 2.5. Observed indicators

#### 2.5.1. Evaluation of main therapeutic effects.

Recording the preoperative dysmenorrhea VAS scores and the corresponding postoperative VAS scores at the moment of operation completion, and at 5, 10, 30, and 60 minutes after the operation completion, respectively (VAS score numbers were assigned by the doctors according to the scale marked by the patients on the VAS ruler, from 0 indicating painless to 10 indicating a most severe, unbearable pain).

#### 2.5.2. Safety parameters.

The preoperative vital signs and the corresponding postoperative vital sign changes at the moment of operation completion, and at 5, 10, 30, and 60 minutes after the operation completion, respectively, including HR, BP, RR, and SPO_2_ were recorded.

Postanesthesia recovery scores were obtained 10 minutes after the completion of the operation using the Modified Alderete score system from the following 5 parameters: level of activity, respiration, circulation, consciousness and blood oxygen saturation. A telephone follow-up visit 1 day after the operation collected report of any discomfort feeling from the patients.

#### 2.5.3. Efficacy index.

Propofol dose (overall dose, loading dose and supplementary dose), ephedrine dose and atropine dose, rescue analgesic drugs were recorded, and the time needed for eyelash reflection disappearance calculated from the beginning of the drug delivery, the operation starting time and completion time, and the postoperative wake-up time (recovery time of orientation ability) were measured using a stopwatch. The satisfaction degrees of the operator, the anesthesiologist, and the patients were rated in a 10-degree system with 0 to 3 denoting inferior, 4 to 7 good, and 8 to 10 excellent.

#### 2.5.4. Adverse events.

Body movement (BM) reflection (BM grading (0/1/2): grade zero for no BM; grade 1 for ordinary BM: movement of toes, hands, distal small joints and other BM without affecting the operation; grade 2 for serious BM including movement of major joints that needs to be limited by holding, and other movements perturbing the operation): whenever grade 2 BM occurs during the operation, supplementary drugs are given. For grade 1 BM occurring in the cervix dilatation at the outset of operation, additional dose of drugs is given; For grade 1 BM occurring after embryo curettage, no supplementary drugs should be given).

##### 2.5.4.1. Incidence of respiratory depression.

Respiratory depression is defined by a mask oxygen inhalation, SPO_2_ ≤ 92% or a respiratory frequency < 8/min. In case the SPO_2_ ≤ 92% and lasting for >30 seconds, the low jaw of the patient is lifted up to improve ventilation; If the SPO_2_ ≤ 90%, a simple airbag mask is used to help the respiration.

#### 2.5.5. Other adverse events recorded including.

Emergence agitation (a numeric degree of emergence agitation is assigned based on the following grading standards: grade 1 sleeping; 2 consciousness; 3 agitation, crying and screaming; 4 unable to be comforted, ceaseless crying and screaming; 5 serious agitation, disorientation.), nausea and vomiting (based on WHO scoring standard (1/2/3/4): 1 no nausea and vomiting; 2 slight nausea and abdominal discomfort without vomiting; 3 apparent nausea and vomiting without gastric content vomiting out; 4 serious vomiting with gastric contents vomiting out that needs to be controlled by medication), dizziness (yes/no), sweating (yes/no), skin itching (yes/no), urine retention (yes/no), erythema and other skin allergies (yes/no).

### 2.6. Statistical analysis

Two-sided tests were adopted in all statistical analyses, and a *P* value ≤0.05 is considered to have statistical significance. The continuous variables were analyzed by variance analysis/Wilcoxon rank sum test according to their normality and homogeneity to describe the average value, standard deviation/median value and rank average value for each group. In case, there are statistically significant differences between the groups, pairwise comparisons were conducted to describe average value difference/rank difference and standard error. For comparison with the control group, Dunnet method was used, while comparisons with other oxycodone dose groups adopted LSD method. Variables in analysis of variance was employed, and in comparing the differences between the groups by controlling gestational variables, single factor linear model was applied for analyzing the variables to describe the mean value and standard deviation of each group. Categorical variables were used to characterize each group’s ratio, and chi-square test used to compare the differences between the groups. However, with an expectation frequency smaller than 5, Fisher exact probability test was then employed for the comparisons. When statistically significant differences were found, chi-square test was applied to conduct pairwise comparison. The VAS scores, intraoperative, and postoperative vital signs were the measured data at each time point, and repeated measurements were used to analyze and compare the differences between different time points and different groups, with the gestational week as the covariant to be controlled.

## 3. Results

**Figure 1.**
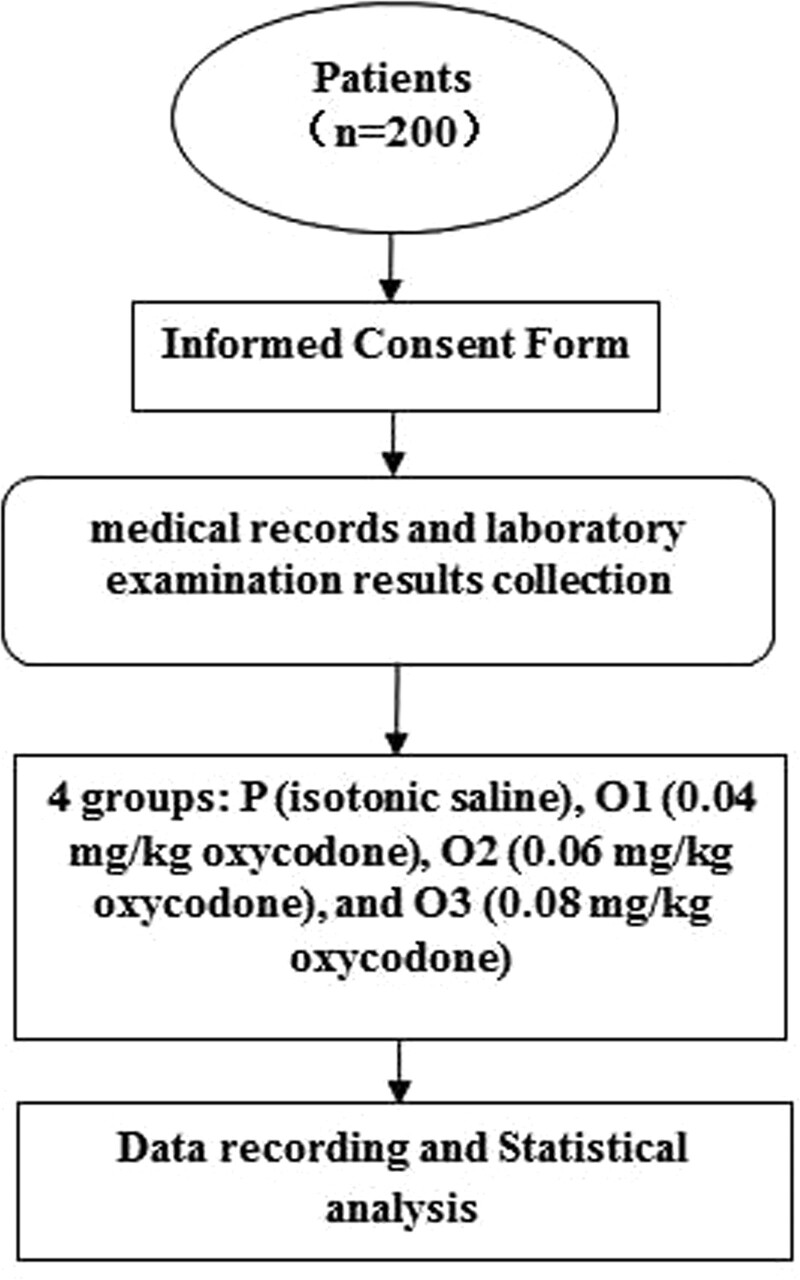
The flow diagram of the study.

Two hundred patients fulfilling the inclusion standards were chosen from a total of 1306 patients of negative pressure aspiration. General conditions of the patients in the 4 groups are listed in Table 1. The times representing the operation efficiency are given in Table 2. The flow diagram of the study was shown in Figure 1.

**Table 1:** Demographic characteristics of patients in the 4 groups.

Group	Number of cases	Age	Body weight (kg)	Height (cm)	BMI (Kg/m^2^)	Gestational weeks	
						Median	Rank average
P	50	27.02 ± 3.72	54.01 ± 6.14	162.10 ± 4.95	20.51 ± 1.66	6.00^a^	81.28
O1	50	26.20 ± 3.90	56.86 ± 8.60	161.90 ± 4.55	21.58 ± 2.97	7.00	123.14
O2	50	27.28 ± 2.97	55.41 ± 10.31	162.80 ± 5.10	20.83 ± 3.17	7.00	99.01
O3	50	25.74 ± 3.49	54.95 ± 7.16	161.90 ± 4.52	20.88 ± 2.33	7.00	98.57

Note: a denotes comparisons of P group with the oxycodone groups under the gestational week conditions. With statistically significant difference, *P* ≤ .001.

**Table 2:** Operation times in the 4 groups.

Group	Eyelash reflection disappearance Time, s	Operation starting time, s	Operation ending time	Anesthesia recovery time		Total duration of Operation	
				Median	rank average	Meian	rank average
P	95.00 ± 7.61	97.94 ± 8.75	301.36 ± 61.94	46.5	102.50	359.00	97.01^a^
O1	93.24 ± 9.74	97.36 ± 11.22	331.42 ± 54.79	55.5	107.13	407.00	115.50
O2	95.84 ± 11.59	96.90 ± 16.22	324.40 ± 52.77	72.5	105.59	411.50	106.88
O3	92.88 ± 10.58	94.82 ± 11.44	309.80 ± 62.40	34.5	86.78	367.00	82.61*

* denote a comparison with the oxycodone groups. With statistically significant difference.

### 3.1. Postoperative VAS scores

The postoperative VAS score shows a decline tendency in the oxycodone groups as compared with group P, with groups O2 and O3 most obvious. The VAS score variation in different groups at each time point has statistical significance (*P* < .01), as shown by the fact that while there is no statistical difference between O1 group and P group, groups O2 and O3 have significant differences of VAS scores from group P (*P* < .01), and the difference between group O3 and O1 is also significant (*P* < .01). They are shown in Tables 3 and 4.

**Table 3:** VAS scores at various time points in the 4 groups.

Time	Group				In the group		Between the groups	
	P	O1	O2	O3	*F*	*P*	*F*	*P*
Before operation	512 ± 1.22	5.30 ± 1.25	5.32 ± 1.28	5.62 ± 1.19	2.172	.081	8.756	.000*
At the moment of anesthesia recovery	1.84 ± 2.08	0.86 ± 1.53	0.82 ± 1.51	0.32 ± 0.79				
5 min	2.62 ± 1.77	2.62 ± 2.01	1.84 ± 1.53	1.68 ± 1.35				
10 min	2.56 ± 1.68	2.54 ± 1.85	1.56 ± 1.42	1.16 ± 1.13				
30 min	1.78 ± 1.09	1.60 ± 1.44	0.94 ± 1.35	0.64 ± 0.85				
60 min	1.50 ± 1.11	0.88 ± 1.15	0.70 ± 1.28	0.46 ± 0.84				

With gestational week adjustment.

*P < 0.05.

**Table 4:** Comparison of the VAS scores in the oxycodone groups and in P group.

Group	Group for comparison	Mean difference	Standard error	*P*
P	O1	0.27	0.20	0.387
	O2	0.71	0.20	0.001*
	O3	0.92	0.20	0.000*

*P < 0.05.

a denotes a comparison with the O1 group with statistical difference.

### 3.2. Intraoperative HR, BP, and respiratory frequency

**Figure 2.**
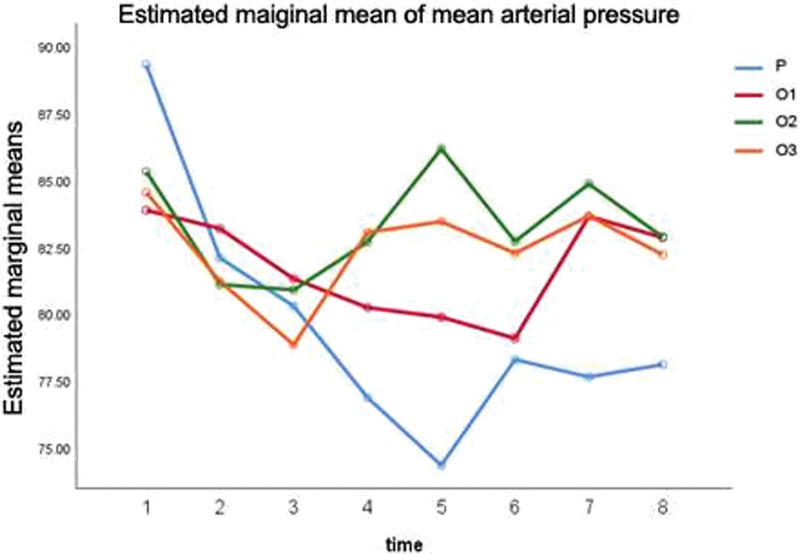
The intraoperative BP (mean arterial pressure).

The intraoperative HR show a decline tendency in all 4 groups, with the differences between group P and groups O1, O3 being statistically significant (*P* < .05), see Tables 5 and 6. However, the intraoperative BP has no statistical meaning, see Table 7 and Figure 2. At each time point, the intraoperative respiratory frequencies of groups O1, O2, and O3 are clearly higher than in group P, and the differences are significant (*P* < .01), see Table 8.

**Table 5:** Comparison of intraoperative HR at different time points and groups.

Time	Value	*P*	O1	O2	O3	Time		Group		Time * Group	
						*F*	*P*	*F*	*P*	*F*	*P*
0	Mean	89.05	76.23	78.44	75.88	12.533	.000	4.093	.010	0.952	.525
	Standard deviation	15.43	6.56	9.38	12.65						
1	Mean	82.50	73.86	75.17	71.50						
	Standard deviation	12.56	6.41	9.91	8.61						
2	Mean	77.25	70.31	71.56	70.75						
	Standard deviation	11.20	5.92	9.98	9.06						
3	Mean	74.55	71.08	70.67	68.42						
	Standard deviation	12.38	5.96	9.01	8.27						
4	Mean	75.20	69.15	71.00	71.29						
	Standard deviation	10.38	5.84	7.27	18.75						
5	Mean	75.45	67.46	71.06	67.58						
	Standard deviation	10.40	5.62	10.91	11.17						
6	Mean	77.35	72.15	73.11	69.88						
	Standard deviation	10.23	5.06	9.00	9.17						
7	Mean	75.85	72.08	74.11	71.38						
	Standard deviation	9.22	5.01	10.41	7.48						

**Table 6:** Comparisons of intraoperative HR.

Group	Group for comparison	Rank difference	Standard error	*P*
P	O1	6.92	2.70	.034*
	O2	5.26	2.46	0.093
	O3	7.57	2.30	.004*

*P < 0.05.

**Table 7:** Comparison of intraoperative mean arterial pressure at different time points and groups.

Time	Value	P	O1	O2	O3	Time		Group		Time * Group	
						*F*	*P*	*F*	*P*	*F*	*P*
0	Mean	88.68	87.78	88.45	85.19	4.661	.000	1.007	.395	1.954	.010
	Standard deviation	9.60	10.05	9.94	8.35						
1	Mean	79.02	80.47	79.86	80.46						
	Standard deviation	9.57	11.16	11.97	9.20						
2	Mean	78.91	82.04	81.91	77.28						
	Standard deviation	9.67	12.99	14.77	9.97						
3	Mean	82.05	85.35	85.97	85.63						
	Standard deviation	11.49	10.29	12.12	12.71						
4	Mean	80.70	85.04	89.20	85.32						
	Standard deviation	11.65	8.85	10.17	11.89						
5	Mean	80.94	85.74	84.67	83.78						
	Standard deviation	12.31	9.75	10.42	10.38						
6	Mean	77.63	85.16	85.66	83.86						
	Standard deviation	13.61	6.21	8.97	6.76						
7	Mean	78.10	82.85	82.87	82.21						
	Standard deviation	9.04	3.71	8.81	5.54						

**Table 8:** Comparisons of intraoperative respiratory frequencies.

Group	Group for comparisom	Rank difference	Standard error	*P*
P	O1	−3.85	0.65	.000*
	O2	−3.31	0.60	.000*
	O3	−2.97	0.56	.000*

### 3.3. Incidence of postoperative hypotension and respiratory depression

**Figure 3.**
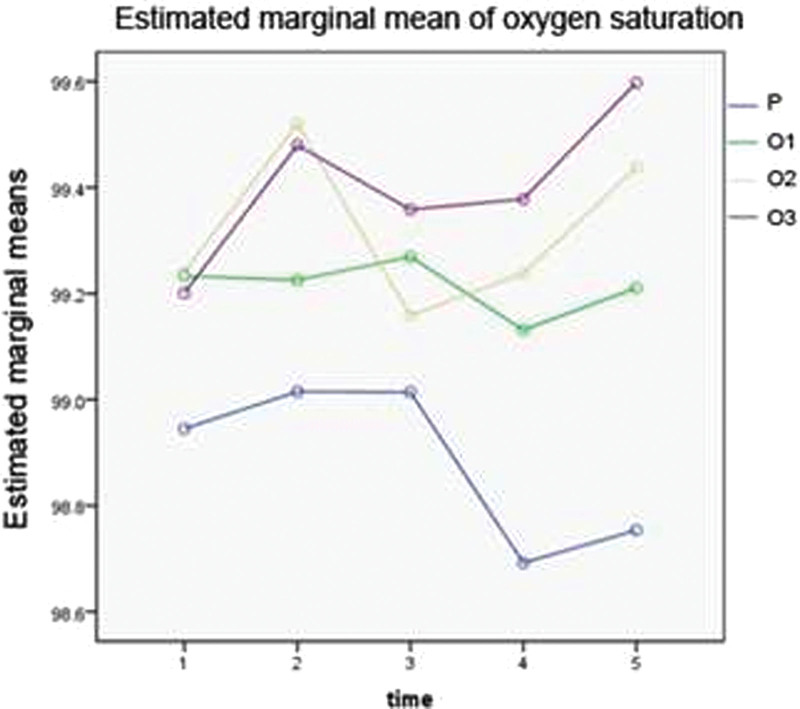
The estimated marginal means of the pulse oxygen saturation. Covariates occurring in the model were estimated at a gestational week of 6.710 *(at the following value: gestational weem 6.710*).

The postoperative SPO_2_ in group P is lower than in groups O2 and O3, with the differences of statistical meaning (*P* < .05), see Table 9 and Figure 3. The incidence of postoperative hypotension in group P is higher than in groups O1, O2, and O3, the differences, however, have no statistical meaning, see Table 10. Group P has 10 cases of respiratory depression (20%), apparently higher than in groups O1 (1 case, 2%), O2 (zero case), and O3 (2 cases, 4%), with the differences being statistically significant (*P* < .05), see Table 11.

**Table 10:** Incidence of postoperative hypotension at different time points, n (%)

Group	0 min	5 min	10 min	30 min	60 min
P	8 (16.0)	7 (14.0)	8 (16.0)	5 (10.0)	4 (8.0)
O1	2 (6.0)	3 (6.0)	3 (6.0)	4 (8.0)	2 (4.0)
O2	5 (10.0)	3 (6.0)	4 (8.0)	5 (10.0)	5 (10.0)
O3	4 (8.0)	2 (4.0)	3 (6.0)	1 (2.0)	2 (4.0)
*P*	0.255	0.319	0.316	0.403	0.613

*P* values were obtained by Fisher Exact Test.

**Table 11:** Comparisons of incidence of respiratory depression.

Group	Group for comparison	Chi-square (χ^2^)	*P*
P	O1	8.274	.004*
	O2	11.111	.001*
	O3	6.061	.014*

*P < 0.05.

**Table 9:** Comparisons of postoperative oxygen saturation of the oxycodone groups with group P.

Group	Group for comparison	Rank difference	Standard deviation	P value
P	O1	−0.27	0.17	0.117
	O2	−0.41	0.17	0.019*
	O3	−0.49	0.17	0.005*

### 3.4. Postanesthesia recovery score

Postanesthesia recovery score was estimated 10 minutes after the finish of the operation with modified Alderete score from the following aspects: activity, respiration, circulation, conciseness, and oxygen saturation. In telephone, follow-ups carried out 1 day after the operation, no uncomfortable symptoms were reported from all 4 groups, with the modified Alderete scores uniformly higher than 9. Postoperative activity, respiration, and oxygen saturation have a score of 2 for all the 4 groups. For circulation, the number of cases receiving a score of 1 differs in the 4 groups: 7 in group P, 6 in group O1, 8 in group O2, and 7 in group O3. 3 cases receiving a conciseness score of 1 in group P. These comparisons gave no significant differences. The *P* values were obtained by Fischer exact test and were reevaluated before leaving the clinic room as having a 10 point score for the 4 groups, see Table 12.

**Table 12:** The total Alderete score (score) n (%).

Group	1	2	Chi-square (χ^2^)	*P*
P	7 (14.0)	43 (86.0)	0.332	.954
O1	6 (12.0)	44 (88.0)		
O2	8 (16.0)	42 (84.0)		
O3	7 (14.0)	43 (86.0)		

### 3.5. Analysis of the propofol dose and body movement

The propofol loading dose and overall dose per kilogram body weight in groups O1, O2, and O3 are all lower than in group P, with the difference between groups O2, O3, and group P having statistical meaning (*P* < .01), see Table 13. The proportion of patients receiving additional injection of propofol is 62% in group P, while this proportion in groups O1, O2, and O3 is 46%, 30%, and 16%, respectively, all lower than in group P, with the difference between group O3 and group P having statistical meaning (*P* < .05), see Table 14. The incidence of BM in group O3 is lower than in groups P, O1 and O2, and the differences are significant (*P* < .01), see Table 15.

**Table 13:** Propofol loading dose and per kilogram body weight loading dose.

Group	Propofol loading dose, mg			Propofol per kilogram body weight loading dose, mg				
	Mean ± standard deviation	Mean difference	*P*	Median	Rank mean	Rank difference	Standard deviation	*P*
P	120.50 ± 17.04			2.10	127.53			
O1	112.90 ± 15.90	7.60	0.009*	2.00	100.22	27.31	10.66	0.062
O2	109.68 ± 14.81	10.82	0.000*	2.00	93.39	34.14	10.66	0.008*
O3	108.16 ± 14.70	12.34	0.000*	2.00	80.86	46.67	10.66	0.000*

*P < 0.05.

**Table 14:** Number and percentage of patients with propofol supplementation and supplementary dose.

Group	Propofol supplementation		Propofol supplementary dose, mg			
	Yes, n(%)	No, n(%)	Median	Rank difference	Standard deviation	*P*
P	31 (62.0)^a^	19 (38.0)	36.23			
O1	23 (46.0)	27 (54.0)	48.39	−12.16	5.96	.248
O2	15 (30.0)	35 (70.0)	44.00	−7.77	6.81	1.000
O3^a^^,^^b^	8 (16.0)	42 (84.0)	13.38	22.85	8.59	.047*

*P < 0.05.

a denotes a comparison with the O1 group with statistical difference.

b denotes a comparison with the O2 group with statistical difference.

**Table 15:** Incidence of body movement, n (%).

Group	Grade 0	Grade 1	Grade 2	*P*
P	17 (34.0)	16 (32.0)	17 (34.0)	
O1	19 (38.0)	17 (34.0)	14 (28.0)	0.806
O2	22 (44.0)	16 (34.0)	12 (24.0)	0.472
O3^a^^,^^b^	36 (72.0)	5 (10.0)	9 (18.0)	0.000*

*P < 0.05.

a denotes comparison with group O1 with statistical difference.

b denotes comparison with group O2 with statistical difference.

### 3.6. The use of uterus dilatation bar, and the level of difficulty of the uterus dilatation

Shorter time was needed for the uterus dilatation for group O3 than for groups P and O1, and the differences are of statistical significance. In groups O3 and O2, the use of uterus dilatation bar is less than in groups P and O1. In groups O1, O2, and O3, the sizes of uterus dilatation bar first time used by clinicians are smaller for groups O1, O2, and O3 than for group P, the differences being significant (*P* < .01). The percentage of uterus expanding difficulty in group P is 26%, higher than in the oxycodone groups as estimated by the clinicians, and the differences are significant (*P* < .01; see Tables 16 and 17).

**Table 16:** Number of uterus expanding rod and the smallest size of uterus expanding rod in the first use.

Group	Number of uterus dilatation bar			smallest size of uterus dilatation bar in the first use		
	Median	Rank mean	*P*	Mean ± standard deviation	*F*	*P*
P	7.00	123.90		4.44 ± 0.40	6.180	
O1	6.00	103.41	.067	4.69 ± 0.40		.005*
O2	6.00	87.98	.001*	4.69 ± 0.51		.005*
O3	6.00	86.71	.001*	4.83 ± 0.44		.000*

*P < 0.05.

**Table 17:** Uterus expanding time and difficulty of Uterus expanding judged by the clinicians.

	Uterus expanding time/second			Uterus expanding difficulty judged by the clinicians			
	Median	Rank mean	*P*	Easy	Fairly easy	Difficult	*P*
P	30.50	114.21		21 (42.0)	16 (32.0)	13 (26.0)	
O1	32.50	112.89	.909	36 (72.0)	14 (28.0)	0 (0.0)	.000*
O2	30.00	94.40	.086	37 (74.0)	9 (18.0)	4 (8.0)	.004*
O3	30.00	80.50	.003*	44 (88.0)	5 (10.0)	1 (2.0)	.000*

*P < 0.05.

### 3.7. Use of concomitant drugs

For bradycardia, 0.5 mg atropine was administered. In case of a systolic pressure lower than 80 mm Hg, 5 to 10 mg ephedrine was delivered. In group P, 2 cases were given astropine and 7 cases were given ephedrine as concomitant drug, and 21 cases had VAS score ≥ 4 (42%). In group O1, 1 case received astropine and 2 cases received ephedrine as concomitant drug, with 15 cases having VAS score ≥ 4. In group O2, 1 case astropine, 1 case ephedrine, and 4 cases with VAS score ≥ 4. In group O3, 1 case ephedrine and 1 case with VAS score ≥ 4. The number of cases with VAS score ≥ 4 in groups O2 and O3 are less than in group P, with significant differences (*P* < .05).

### 3.8. Other advert events

In the classification of emergence agitation, rank mean values in the conciseness grade (grade 2) in groups O3, O2, and O1 are 117.56, 103.20, and 98.16, respectively, all higher than the 83.08 in group P, but only the difference between group O3 and group P is statistically meaningful (*P* < .01). The incidences of nausea and vomiting are 4 cases in group P (8%), 4 cases in O1 (8%), 5 cases in O2 (10%), and 3 cases in O3 (6%), with no statistically significant differences. The incidences of dizziness are 5 cases in group P (10%), 2 cases in O1 (4%), 2 cases in O2 (4%), and 5 cases in O3 (10%).

### 3.9. Satisfaction analysis

For the median value of 9 in O3 group evaluated by the surgeons, the satisfaction average rank is 137.31; for the median value of 10.00 in O3 group evaluated by the anesthesiologists, the average rank is 142.99; for the median value of 9.00 in O3 group evaluated by the patients, the average rank is 120.99. The satisfaction average rank values for O3 group from surgeons, anesthesiologists, and patients are obviously higher than the median value and average rank for group P, but the differences are not significant, see Table 18.

**Table 18:** Satisfaction scores from surgeon, anesthesiologists and patients.

	Surgeon		Anesthesiologist		Patient	
	Median	Rank average	Median	Rank average	Median	Rank average
P	8.00	81.18	7.50	70.48	8.00	76.38
O1	9.00	93.13	8.00	93.26	9.00	92.01
O2	9.00	90.38	9.00	95.27	9.00	112.62
O3	9.00	137.31	10.00	142.99	9.00	120.99
*P*	.491		.420		.467	

## 4. Discussion

The outpatient negative pressure aspiration is typically associated with intense pain produced in the processes of cervical dilatation and suction curettage of the uterus wall, a visceral pain. These operations also stimulate the vagus nerves to induce release of large amount of acetylcholine, which may lead to artificial abortion syndrome. The use of outpatient general anesthesia in negative pressure aspiration substantially reduce the probability of artificial abortion syndrome.^[5]^ In this case, the operation is routinely carried out by anesthesia with propofol alone or with propofol combined with opioid analgesics.^[6]^ Propofol is the most commonly used intravenous anesthetic in modern anesthesia. However, due to the relatively poor analgesic effect of propofol, patients usually experienced BM due to pain stimulation during operation, which increasing the risk of operation. Strong stimulation such as cervical dilatation and uterine wall suction during negative pressure uterine suction with propofol alone often needs deep sedation, while large dose of propofol will lead to double inhibition of respiratory system and circulatory system and delay of wake up. Therefore, propofol combined with opioids can not only effectively reduce the dosage of propofol and the incidence of adverse reactions, but also provide good intraoperative and postoperative analgesia and improve the overall satisfaction of patients.

Propofol is delivered either by single injection or by target controlled infusion. However, propofol is not a strong analgesic, and strong stimulation such as cervix dilatation often need to be antagonized by deep sedation, leading to a frequent occurrence of deep respiratory depression and circulatory depression. At the same time, fentanyl, remifentanil, and sufentanil are specific μ opioid receptor agonist, they have poor analgesic effect toward uterine contraction pain and are apt to induce such side effects as nausea, vomiting, and respiratory depression. Raeder showed that, among abortion patients with general anesthesia, 25% suffered from apnea and 67% suffered from postoperative pain.^[7]^ Visceral pain is characterized by diffusivity and lacking locality. Its pathophysiology and basic neurobiological mechanism are still not fully understood.^[8]^ In recent years, some research attention has been paid to the mechanism of pain regulation by sex hormones. It is shown that rapid increase of estrogen may inhibit the analgesic function of μ-opioid receptor agonist, probably by altering connection of G protein with the corresponding effector system.^[9]^ Dysmenorrhea refers to a group of signs and symptoms of general malaise occurring before, during or after the menstrual period, characterized mainly by lower abdomen spastic pain accompanying by headache, fatigue, dizziness, nausea, vomiting, diarrhea, abdominal distension, backache, and so on. Dysmenorrhea is quite common among young females and about half of them experience this symptom during their menstrual period, mostly manifested as a paroxysmal colic pain lasting for 12 to 24 hours with concomitant headache, dizziness, breast distention, frequent urination, constipation or diarrhea, insomnia, irritability, and so on. In some serious cases, pale face, cold sweat, cold extremities, nausea, vomiting or even syncope of the patients may be observed.^[9]^ Serious dysmenorrhea severely hampers the sufferers’ normal life and work. For the dysmenorrhea patients, their postoperative uterine contraction pain may be worthened by the prostaglandin generation, smooth muscle spasm and their nervous mood brought about by the periodic pain. Studies of Chen et al show that, under propofol anesthesia, the VAS scores at the moment of, and at 15 minutes and 30 minutes after the anesthesia recovery for the dysmenorrhea and nondysmenorrhea groups of patients are significantly different, with the former feeling higher degree of pain.^[3]^ Geng et al studied the factors relevant to uterine contraction pain after abortion and conducted a Pearson correlation analysis in regard to age, body weight, dysmenorrhea and the postoperative uterine contraction pain.^[10]^ They found that. when using the same anesthetic, there was a close correlation between the degree of uterine contraction pain and the dysmenorrhea history with a correlation coefficient of 0.651. The higher the dysmenorrhea VAS score, the higher the VAS score of postoperative uterine contraction pain, with a positive correlation. The drugs presently used for the treatment of dysmenorrhea include NSAIDS, magnesium sulfate, inhalation anesthetics, ketamine, opioids, and so on; all of them have shortcomings as slow action, rather weak analgesic effect, narrow therapeutic window easily leading to drug poisoning, inducing nausea and vomiting, onset of spiritual symptoms, and respiratory depression. Negative pressure aspiration is a rapid operation with fast patient turnover and requires anesthetics with quick action and metabolism, definite analgesic effect, and complete recovery.^[1]^

As a semisynthesized opioid, oxycodone serves as a dual agonist of μ and κ receptors. Known research results show that oxycodone is a good analgesic treating visceral pain.^[4]^ This is owing to the fact that oxycodone has strong analgesic effect with an onset time of 2 to 3 minutes and an elimination half-life of 3 to 5 hours. The side effect of oxycodone is dependent on the dosage. The metabolism products of oxycodone are not active or have only little activity, with less possibility to induce advert events. As a matter of fact, the application of oxycodone leads to less agitation and almost no respiration depression and Inhibition of gastrointestinal peristalsis.^[4]^ The oxycodone plays the analgesic effect mainly by acting on the central nervous system and smooth muscle. By acting on the central nervous system, the analgesic effect is achieved through activating the μ opioid receptor on the cell membrane of the presynaptic nerve ending cells to cut down or block the signal transmission from C fiber to spinal dorsal horn neurons. In the meanwhile, the analgesic effect realized by acting on the organs of smooth muscles through binding to the κ receptor is stronger than that of the traditional μ-receptor agonists.^[10,11]^ The low affinity of oxycodone toward μ receptor also resulted in low incidences of respiratory depression, nausea and vomiting.^[12]^ In this study, the incidence of respiratory rate decrease in each dose oxycodone group was lower than that in propofol group, and the postoperative oxygen saturation in propofol group was lower than that in 0.06 mg/kg oxycodone group. The activation of κ receptor inhibits central respiratory activity, but it also antagonizes respiratory depression mediated by μ receptor, which explains why oxycodone causes less respiratory depression than fentanyl.^[11,12]^ Considering the safety of anesthesia from the perspective of respiratory depression, the anesthesia management of negative pressure uterine aspiration is better in propofol combined with oxycodone group than in propofol alone. These results show that oxycodone may have substantial advantages in postoperative early stage pain-relief.^[4]^

It was reported that oxycodone injection significantly increased the degree of satisfaction of the patients receiving negative pressure aspiration.^[13]^ In our study, we have recorded the satisfaction estimations from the surgeons, the anesthesiologists, and the patients. In group O3, the rank average at a median value 9 for the surgeons is 137.31, while that at a median 10 for the anesthetists is 142.99 and is 120.99 at a median 9 for the patients without knowing their group assignment. The satisfaction degrees of the surgeons, anesthetists and patients in group O3 are all higher than in group P as shown by the obviously higher average ranks in group O3 than group P. The differences are, however, not statistically significant, and satisfaction estimation based on larger sample size is expected.

Intravenous anesthesia has no ideal effect for the cervix relaxing, especially for patients of first pregnancy. To reduce pain and injury, surgeons sometimes apply artificially synthesized prostaglandin E1 analogues, such as misoprostol, prior to the operation for cervix softening and dilatation.^[14]^ In a study on the influence of oxycodone effect-site concentration on cervix dilatation in propofol-controlled artificial abortion, Xie and his coworkers found that a 0.01 mg/kg dose of oxycodone led to a good relaxation of cervix opening to allow the passage of no. 6 dilatation bar for 80% of patients, and this effectively reduced the propofol EC50, decreasing the propofol dose. They further found that, the higher the oxycodone dose was, the stronger effect could be achieved within the dose range they have used. Therefore, the combined use of oxycodone and propofol was shown advantageous by leading to convenient operation procedures, good analgesic performance and more stable hemodynamics. We found that the cervical dilation time of 0.08 mg/kg oxycodone group was shorter than that of 0.04 mg/kg oxycodone group and propofol group. The number of cervical dilators in 0.08 mg/kg and 0.06 mg/kg group was less than that in 0.04 mg/kg and propofol group. The smallest cervical dilators used by clinicians for the first time in different doses of oxycodone group were smaller than those in propofol group. Clinicians judged that the difficulty rate of cervical dilatation in propofol group was 26%, which was higher than that in propofol-oxycodone compound group. There are also significant differences in the time required for cervical dilatation. The results show that oxycodone can soften the cervix and is suitable for negative pressure suction anesthesia.

In summary, oxycodone in 0.06 mg/kg and 0.08 mg/kg dose combined with propofol can be safely and effectively used in negative pressure aspiration anesthesia. These compatibility medicines relieve the postoperative uterine contraction pain efficiently with stable intraoperative and postoperative vital signs and relatively less advert events. Especially, the 0.08 mg/kg dose of oxycodone combined with propofol leads to reduced propofol dose, represents a better combination worthy of more extensive clinical applications. Moreover, in the future, a comparative study of propofol combined with other analgesic drugs will be added to see if oxycodone K receptor has a better inhibitory effect on uterine contraction pain after negative pressure uterine aspiration in dysmenorrhea patients. With regard to the dosage grouping of oxycodone, this study shows that oxycodone 0.08 mg/kg has a better effect on intraoperative efficacy and inhibition of postoperative uterine contraction pain. Considering that a larger dose of oxycodone will increase dizziness or prolong recovery time, this study did not do a study on a larger dose of oxycodone.

## Author contributions

Yi-Nan Wang, Ying Feng, Fei Yao, Lu-Wen Zou and Hua Feng acquired and analyzed the patient data. Yi-Nan Wang, Ming-Jun Xu, Xin-Zhong Chen, Ming-Kun Shen and Lei Zhao analyzed and interpreted the patient data. Yi-Nan Wang, Ming-Jun Xu and Jian-Guo Xu were major contributors in designing the study and writing the manuscript. All authors read and approved the final manuscript.

Consent for publication: All data have been approved for publication.

Availability of data and material: The data are available from the corresponding author on reasonable requirement.
